# Epidemiology of ocular pathology in domestic animals: insights from a 20-year retrospective study

**DOI:** 10.3389/fvets.2025.1717392

**Published:** 2026-01-05

**Authors:** Jamile Macedo Garcia, Guilherme da Silva Rogerio, Carlos Alberto Rossatto-Júnior, Lorena Santos Bezerra, Érica Thurow Schulz, Raphael Assis Leandro de Morais, Maria Lucia Zaidan Dagli

**Affiliations:** 1 Experimental Physiopathology Program, Faculty of Medicine, Department of Pathology, University of São Paulo, São Paulo, Brazil; 2 Laboratory of Experimental and Comparative Oncology, Department of Pathology, School of Veterinary Medicine and Animal Science, University of São Paulo, São Paulo, Brazil

**Keywords:** ocular pathology, histopathology, squamous cell carcinoma, eyelid tumors, brachycephalic dog breeds, domestic animals

## Abstract

Ocular pathology holds significant importance in veterinary medicine, providing essential insights into diagnosing and characterizing eye and periocular diseases in animals. This retrospective study aimed to identify and characterize the most frequent lesions affecting the eyes and surrounding structures in domestic animals. A total of 375 ocular and periocular samples, retrieved from the Animal Pathology Service of the Veterinary Hospital at the School of Veterinary Medicine and Animal Science of the University of São Paulo, São Paulo, Brazil, from 2003 to 2022, were analyzed. Most samples came from dogs (64%), followed by cats (15.20%), and horses (12.27%). Neoplastic lesions were the most common (80.53%), especially squamous cell carcinoma (SCC) (20.26%), followed by inflammatory (10.4%) and other lesion types (e.g., hyperplastic, cystic, infectious). Eyelids were the most frequently affected anatomical site. SCC was particularly frequent in the third eyelid of cattle and in the eyelids and conjunctiva of cats and horses. A statistically significant difference was observed between brachycephalic and non-brachycephalic dog breeds, with the former showing fewer neoplastic lesions. Clinical suspicion matched the histopathological diagnosis in 84.1% of cases, although the agreement was moderate according to the Kappa coefficient. Unique findings included a high incidence of meibomian adenocarcinomas and ocular melanoma in dogs, and conjunctival SCC in cats. These results underscore the importance of histopathological evaluation for accurate diagnosis and recommend increased sample submissions to support pathologist training and improve diagnostic accuracy.

## 1 Introduction

Ocular pathology in animals comprises a broad spectrum of inflammatory, degenerative, neoplastic, and traumatic disorders affecting the globe and periocular tissues. Both prospective and retrospective surveys indicate that ocular and periocular lesions are common findings in veterinary diagnostic practice, reflecting the anatomical complexity of the eye and its vulnerability to infectious, immune-mediated, and environmental insults (1, 2). Ocular and periocular neoplasms in animals represent a clinically important albeit relatively uncommon subset of tumors, with potentially serious consequences for vision, ocular integrity, and animal welfare. Although they occur less frequently than neoplasms of skin or mammary gland, ocular tumors remain a meaningful component of veterinary oncologic pathology. Their epidemiology is shaped by host species, breed predisposition, age, environmental exposures (especially ultraviolet radiation), and, in some species, infectious or inflammatory cofactors (2, 3).

Retrospective studies in veterinary pathology are central to defining the epidemiology of ocular lesions in different species. Their primary strength lies in aggregating histopathologic submissions over long time spans. This capacity allows for the estimation of relative frequencies, species and site predilections, and temporal trends (1, 2). However, these studies are inherently subject to biases. They only capture cases submitted for histopathology, and geographic or institutional referral bias may skew the distribution of species or lesion types (2). Nevertheless, such retrospective series remain indispensable for comparative pathology, aiding in risk factor identification and guiding clinical suspicion, diagnostic protocols, and preventive strategies (1, 2).

Recent technological advances permit the direct visualization of structural changes in ocular components, enabling the macroscopic diagnosis of certain conditions (3). Despite these developments, the true incidence and prevalence of ocular lesions in animals are likely underestimated. Many lesions remain clinically unrecognized, are not biopsied, or are misdiagnosed without histologic confirmation. Consequently, only a limited number of samples are submitted for pathological evaluation. The involvement of veterinary pathologists is often restricted to cases exhibiting macroscopic features suggestive of neoplasia (4).

This study aims to identify and characterize the most prevalent ocular and periocular lesions in animals submitted for histopathological evaluation at a veterinary pathology service in São Paulo, Brazil.

## 2 Materials and methods

### 2.1 Data retrieved and inclusion criteria

Histopathological examinations of ocular and periocular samples submitted to the Animal Pathology Service of the Veterinary Hospital at the School of Veterinary Medicine and Animal Science, University of São Paulo (HOVET - FMVZ/USP), in São Paulo, Brazil, were analyzed. This retrospective study covered a 20-year period, from January 2003 to December 2022.

Inclusion criteria encompassed biopsy cases of the globe and its adnexa submitted during the study period, with anatomical location indicated either directly in the registry or inferred from a morphological diagnosis compatible with ocular structures. Collected variables included species, age at the time of examination (in years), breed, sex, anatomical location of the lesion, duration of clinical signs (in days), clinical suspicion, lesion type, morphologic diagnosis, and, when available, etiological diagnosis. Exclusion criteria applied to cases lacking a definitive diagnosis, those containing insufficient or autolyzed material, samples derived from necropsied animals, or material associated with experimental research.

### 2.2 Statistical analysis

Data were organized and tabulated in Microsoft Excel 365, and analyzed using GraphPad Prism 8.0.2 and R 4.5.1. Statistical significance was set at *p* < 0.05 for all tests.

To minimize bias in the analysis of associations between variables, animals with multiple lesions or anatomical sites were represented by their primary lesion, that is, the alteration that prompted the histopathological examination request. This was applied to evaluate the relationships between animal characteristics and lesions. When analyses focused exclusively on lesions, each lesion from animals with multiple lesions was treated as a separate entry.

For statistical purposes, except when comparing neoplastic to non-neoplastic lesions, both lesion type and clinical suspicion were grouped into three categories: neoplastic, inflammatory, and other. The “other” category included hyperplastic, infectious, parasitic, cystic, degenerative, traumatic, vascular, pigmentary, and atrophic lesions. This grouping approach was used because several individual categories contained few cases, which could reduce the statistical power of the analyses. Agreement between clinical suspicion and histopathological diagnosis was assessed using Cohen’s Kappa coefficient.

Associations between categorical variables were tested using the chi-square test (with or without Yates’ correction) or Fisher’s exact test, depending on sample size. Standardized residuals identified the groups that contributed most to significant chi-square results, and Cramér’s V quantified the overall strength of association between variables.

The Anderson-Darling and D’Agostino-Pearson tests were used to assess the normality of the variables “evolution time” and “age.” As the data did not meet the assumptions of normality, nonparametric tests were employed: the Mann–Whitney U test for comparisons between two groups, and the Kruskal-Wallis test for comparisons among more than two groups, followed by Dunn’s multiple comparison test when appropriate.

Outliers in the “age” variable were identified using the Robust Regression and Outlier Removal (ROUT) test, with a false discovery rate (Q) of 1%.

## 3 Results

### 3.1 Case selection and overall frequency of ocular lesions

Between 2003 and 2022, a total of 9,382 cases were submitted for histopathological examination to the Animal Pathology Service of HOVET-FMVZ/USP. Of these, 418 involved the globe and ocular adnexa, representing approximately 4.5% of all cases. Following a review of the examination results, cases that did not meet the inclusion criteria were excluded, resulting in 375 cases for analysis. The main data are summarized in Table 1.

**Table 1:** Summary of ocular and periocular lesions diagnosed between 2003 and 2022 at the animal pathology service (HOVET-FMVZ/USP).

Data analyzed	Categories	*N* (%)	Most frequent lesion types or notes
Species	Canine	240 (64.00%)	Meibomian gland tumors, uveal melanoma
	Feline	57 (15.20%)	SCC (eyelid)
	Equine	46 (12.27%)	SCC (cornea)
	Bovine	15 (4.00%)	SCC (third eyelid)
	Others	16 (4.26%)	
Age (Years)	Range	0.5–30	
	Mean ± SD	9.35 ± 3.999	
Sex	Male	161 (43.16%)	
	Female	212 (56.84%)	Number influenced by felines and bovines
Anatomical site	Globe	103 (27.47%)	Melanoma
	Eyelid	151 (40.27%)	SCC, meibomian tumors
	Third eyelid	60 (16.00%)	SCC (bovine)
	Conjunctiva	28 (7.47%)	SCC (feline)
	Periocular skin	23 (6.13%)	
	Retrobulbar	10 (2.67%)	Malignant tumors
Lesion type	Neoplasia	301 (80.53%)	SCC
	Inflammatory	39 (10.40%)	Acessory glands, conjunctivae
	Hyperplasia	12 (3.20%)	Acessory glands
	Others	23 (5.87%)	
Lesion duration (days)	Range	3–2,187	
	Neoplasia Mean ± SD Median	266.2 ± 384.2 152	
	Inflammation Mean ± SD Median	134.2 ± 177.5 60	
	Other Mean ± SD Median	83.14 ± 71.27 60	

The lesions identified included 301 neoplasms (80.53%), 39 inflammatory lesions (10.40%), 12 hyperplastic processes (3.20%), seven cystic lesions (1.60%), five infectious processes (1.33%), three traumatic lesions (0.80%), two degenerative conditions (0.53%), one case of atrophy (0.27%), one vascular lesion (hemorrhagic) (0.27%), and one pigmentary lesion (0.27%). Additionally, three cases (0.80%) were within normal histological limits.

Among the five infectious cases, two were bacterial infections, one was equine habronemiasis, one was a parasitic granuloma, and one was a papillomavirus-induced tumor. One additional case of myiasis, considered secondary to a primary lesion of undetermined cause, was also recorded. The most frequent lesion was squamous cell carcinoma (SCC), with 76 cases (20.26%), followed by meibomian epithelioma, with 33 cases (8.8%), observed exclusively in dogs.

### 3.2 Species and breed distribution

Samples were received from 240 dogs (64.00%), 57 cats (15.20%), 46 horses (12.27%), 15 cattle (4.00%), four sheep (1.07%), three Amazon parrots (*Amazona aestiva*) (0.80%), two goats (0.53%), and two mules (0.53%). Single cases (0.27% each) were also recorded from a rabbit, an ostrich, a red-eared slider (*Trachemys scripta*), a plain parakeet (*Brotogeris tirica*), and a domestic pigeon (*Columba livia*). One sample (0.27%) did not have the species identified. No statistically significant association was found between species and lesion type (*p* = 0.2902).

The association between breed and lesion type, categorized as neoplastic or non-neoplastic, was analyzed separately for dogs, cats, and horses. A significant association was detected only in dogs, specifically between brachycephalic and non-brachycephalic breeds (*p* = 0.0394) (Table 2), suggesting that the distribution of neoplastic versus non-neoplastic lesions differs between these groups. Among non-brachycephalic dogs, 82.59% of lesions (166/201) were neoplastic, compared with 66.67% (24/36) in brachycephalic dogs. In addition to Fisher’s exact test, a chi-square test with Yates’ correction was applied for a more conservative assessment, and the result remained statistically significant (χ^2^ = 4.87; *p* = 0.048). The calculated odds ratio was 0.42 (95% CI: 0.19–0.93), indicating that brachycephalic dogs had a lower likelihood of developing neoplastic lesions compared to non-brachycephalic breeds.

**Table 2:** Association between brachycephalic and non-brachycephalic breeds, and the odds of developing neoplasia.

Breeds	Cases (neoplasia)	Controls (non-neoplastic)	Total	% Neoplasia	Odds ratio (OR) [95% CI]
Non-brachycephalic	166	35	201	82.6%	Ref.
Brachycephalic	24	12	36	66.7%	0.42 (0.19–0.93)
Total	190	47	237		

Brachycephalic dogs had a significantly lower odds of developing neoplasia (OR = 0.42; 95% CI: 0.19–0.93) compared to the reference group. OR, odds ratio. CI, confidence interval. Ref, reference.

To evaluate whether age acted as a confounding factor in this association, the ages of brachycephalic and non-brachycephalic dogs diagnosed with neoplasia were compared. The mean age of brachycephalic dogs was 9.57 years (media*n* = 10), while the mean age of non-brachycephalic dogs was 9.73 years (media*n* = 10). No significant difference was observed between the groups, as determined by the Mann–Whitney U test (*p* = 0.6633), suggesting that age was not a confounding variable in the observed association.

### 3.3 Anatomical distribution of lesions

**Figure 1:**
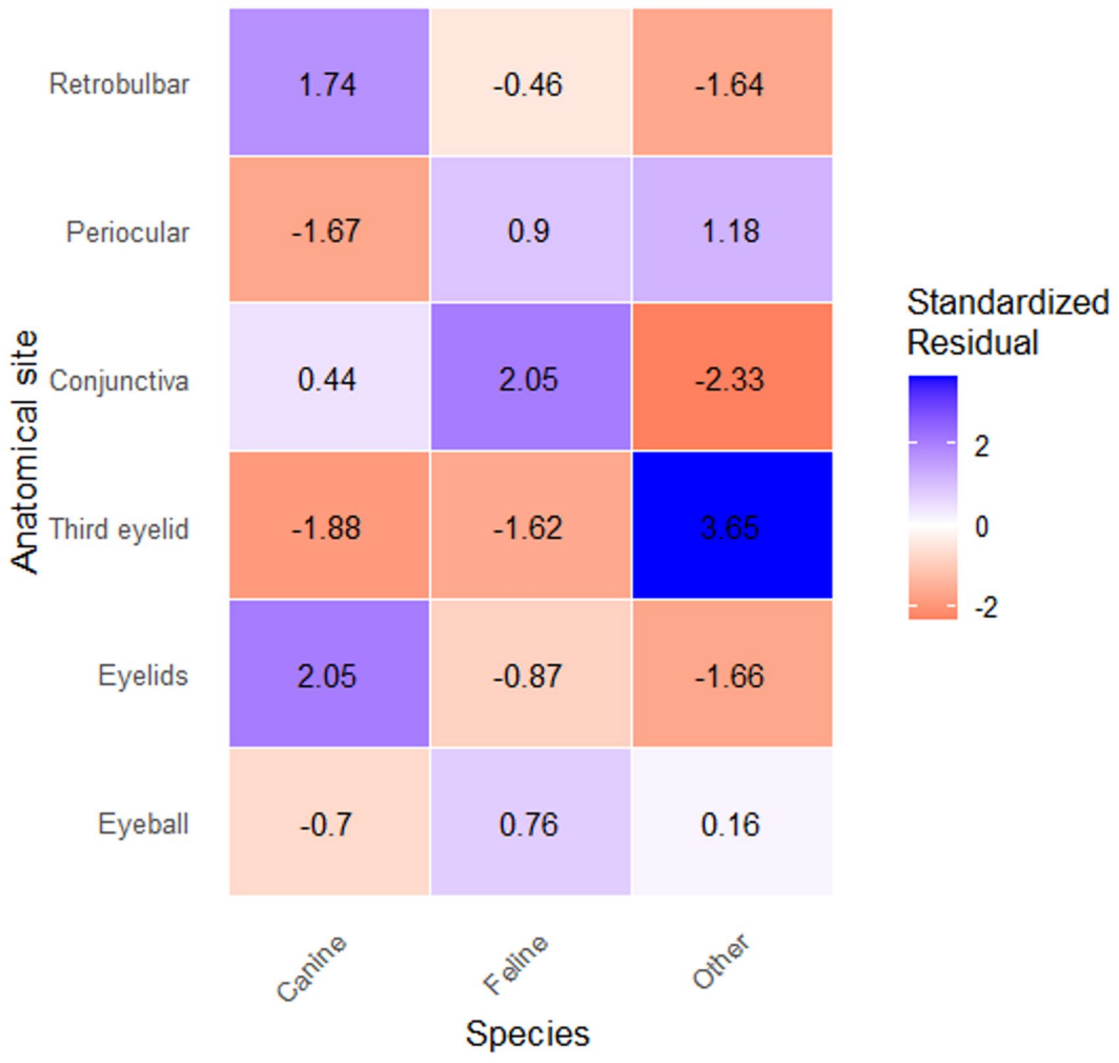
Heatmap displaying the association between anatomical site of ocular lesions and species group. Blue tones indicate a significantly higher frequency than expected (positive residuals), while red tones indicate a significantly lower frequency than expected (negative residuals). White tones indicate frequencies close to the expected. The “Other” group showed a significantly higher frequency of third eyelid lesions (residual = +3.65), and a significantly lower frequency of conjunctival (residual = −2.33) and eyelid (residual = −1.66) lesions than expected. The “Feline” group showed a significantly higher frequency of conjunctival lesions (residual = +2.05).

Regarding anatomical location, and considering only the primary lesion, the following distribution was observed: 151 eyelids (40.27%), 103 entire globes or globe fragments (27.47%), 60 third eyelids (16.00%), 28 conjunctivae (7.47%), 23 periocular skin regions (6.13%), and 10 retrobulbar tissue samples (2.67%). For analysis of the association with species, data were grouped into canines, felines, and others. Further subdivision would have resulted in groups with small sample sizes and reduced statistical power. A statistically significant association was detected (χ^2^ = 28.17; *p* = 0.0017). Standardized residual analysis revealed that the “others” group had a significantly higher frequency of third eyelid lesions than expected (residual = +3.65), while felines exhibited more conjunctival lesions than expected (residual = +2.05). The “others” group showed fewer conjunctival and eyelid lesions than expected (residuals = −2.33 and −1.66, respectively) (Figure 1; Table 3).

**Table 3:** Distribution of ocular and periocular lesions by species and anatomical site, and standardized residuals from the chi-square analysis.

Anatomical location	Canine	Feline	Other	Total
Globe	63 (−0.7)	18 (0.76)	22 (0.16)	103
Eyelid	106 (2.05)	20 (−0.87)	25 (−1.66)	151
Third eyelid	32 (−1.88)	5 (−1.62)	23 (3.65)	60
Conjunctiva	19 (0.44)	8 (2.05)	1 (−2.33)	28
Periocular	11 (−1.67)	5 (0.9)	7 (1.18)	23
Retrobulbar	9 (1.74)	1 (−0.46)	0 (−1.64)	10
Total	240	57	78	375

Positive residuals (> + 1.96) indicate categories contributing positively to the association; negative residuals (< −1.96) indicate categories contributing negatively.

The elevated frequency of third eyelid lesions was primarily due to the number of cattle diagnosed with SCC at this site (12/15). Among these 15 cattle, 14 were Holstein, and one was Girolando, a cross between Holstein and Gir breeds, which exhibited only granulation tissue in the third eyelid.

### 3.4 Distribution considering multiple lesions per animal

**Figure 2:**
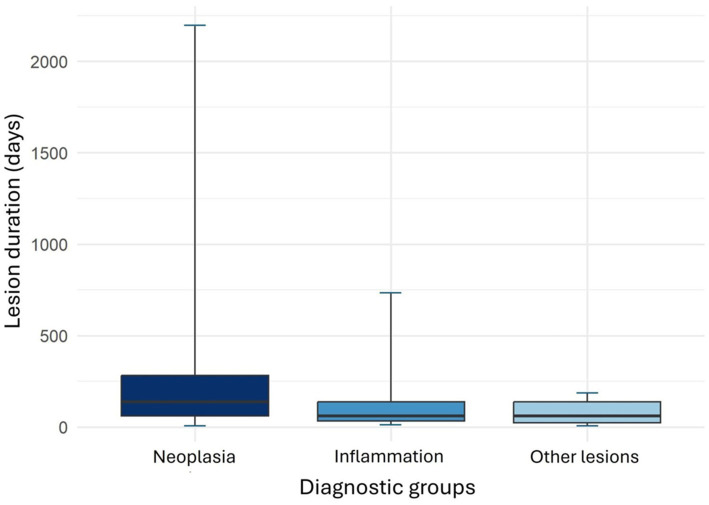
Distribution of ocular lesions by anatomical site and histopathological category. Neoplasms are the predominant lesion type across all anatomical sites, particularly in the eyelid and retrobulbar region.

When accounting for multiple anatomical locations and lesions whithin individual animals, a total of 474 distinct lesions were identified. These included 205 eyelids (43.25%), 120 globes (25.32%), 70 third eyelids (14.77%), 38 conjunctivae (8.02%), 29 periocular skin samples (6.12%), and 12 retrobulbar tissue (2.53%). Among these lesions, 328 were classified as neoplasms (69.20%), 97 as inflammatory (20.46%), 14 as hyperplasia (2.95%), 13 as normal tissue (2.74%), seven as cystic lesions (1.48%), five as infectious diseases (1.05%), three as degenerative (0.63%), three as traumatic injuries (0.63%), two as atrophies (0.42%), one as a pigmentary (0.21%), and one as a vascular lesion (0.21%) (Figure 2). A statistically significant association was found between lesion type and anatomical location (χ^2^ = 22.25; *p* = 0.0139). However, the strength of this association was weak (Cramér’s V = 0.153).

### 3.5 Demographic characteristics

**Figure 3:**
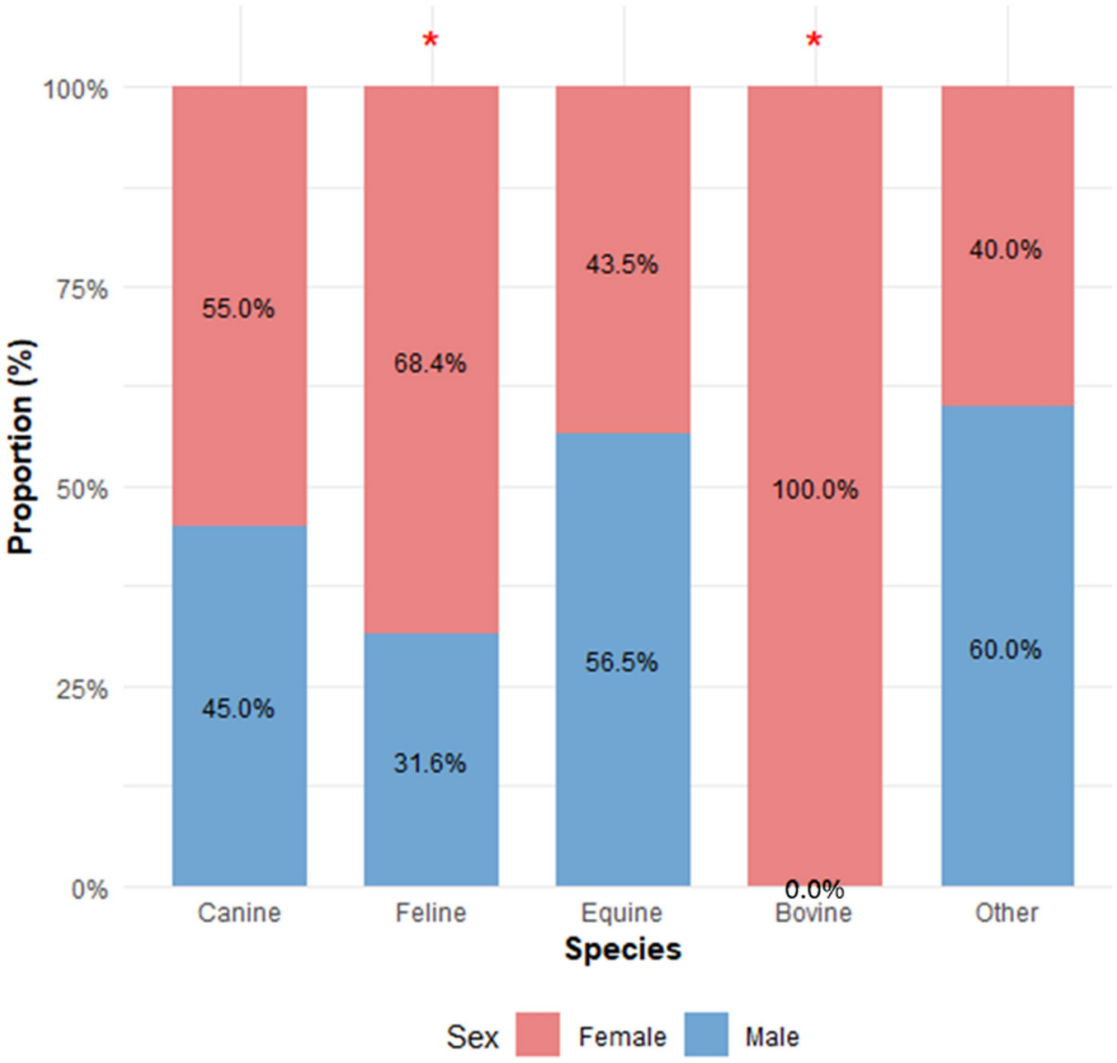
Sex distribution of the animals categorized by species group. The stacked bar graph illustrates the proportion of male and female animals across the species groups (Canine, Feline, Equine, Bovine, and Other), totaling 373 animals where sex was identified. The red asterisk above the bars indicates a statistically significant difference in sex distribution within the cats (68.4% female; *p* = 0.0054) and the cattle (100.0% female; *p* = 0.0001).

Among animals for which sex was identified through referral forms or the hospital’s registration system, 212 were female (56.84%) and 161 were male (43.16%), showing a statistically significant difference in sex distribution (*p* = 0.0083). When analyzed by species, only felines and cattle exhibited similar trends, with a predominance of females: 39/57 felines and all 15 cattle were female (*p* = 0.0054 and *p* = 0.0001, respectively) (Figure 3). However, no significant association was found between sex and lesion type (*p* = 0.8451).

**Figure 4:**
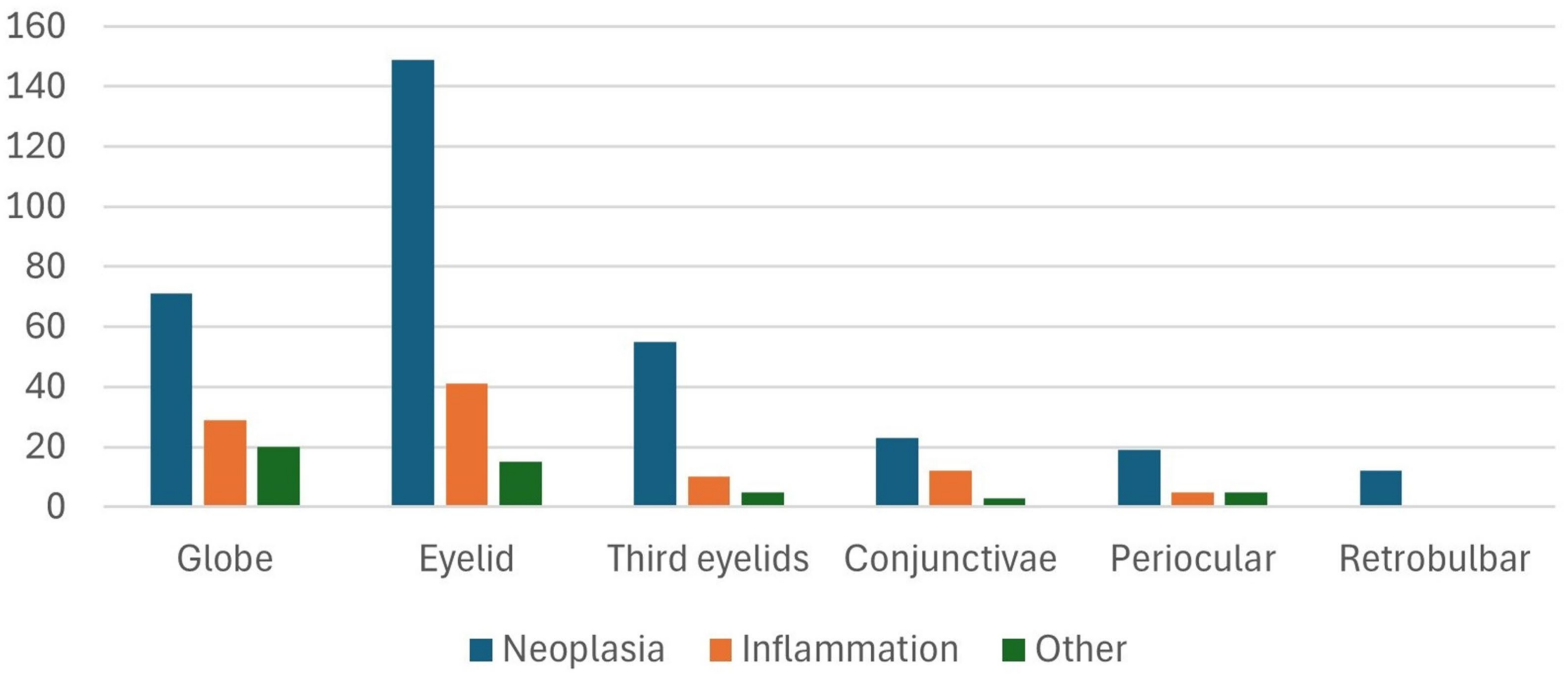
Age distribution of the animals evaluated. Note the two cases considered outliers, one being a 25-year-old horse and the other a 30-year-old parrot. A positively skewed distribution is evident in the graph, caused by the higher number of adult and elderly animals.

Animal ages ranged from 6 months to 30 years, with a mean of 9.35 years (SD = 3.999) and a median of 9 years, indicating a predominance of adult and elderly animals. The Anderson–Darling test for normality yielded *A*^2^ = 1.346 and *p* = 0.0017, confirming deviation from normal distribution. The data exhibited a slight positive skew, primarily influenced by two outliers: a 25-year-old horse and a 30-year-old parrot (Figure 4). Even after excluding these outliers, the distribution remained positively skewed. Analysis of the association between age and lesion type revealed a statistically significant difference (*p* = 0.0004). This difference was most pronounced between animals diagnosed with neoplasms and those in other diagnostic categories, indicating a tendency for older animals to develop neoplastic conditions.

### 3.6 Lesion duration

**Figure 5:**
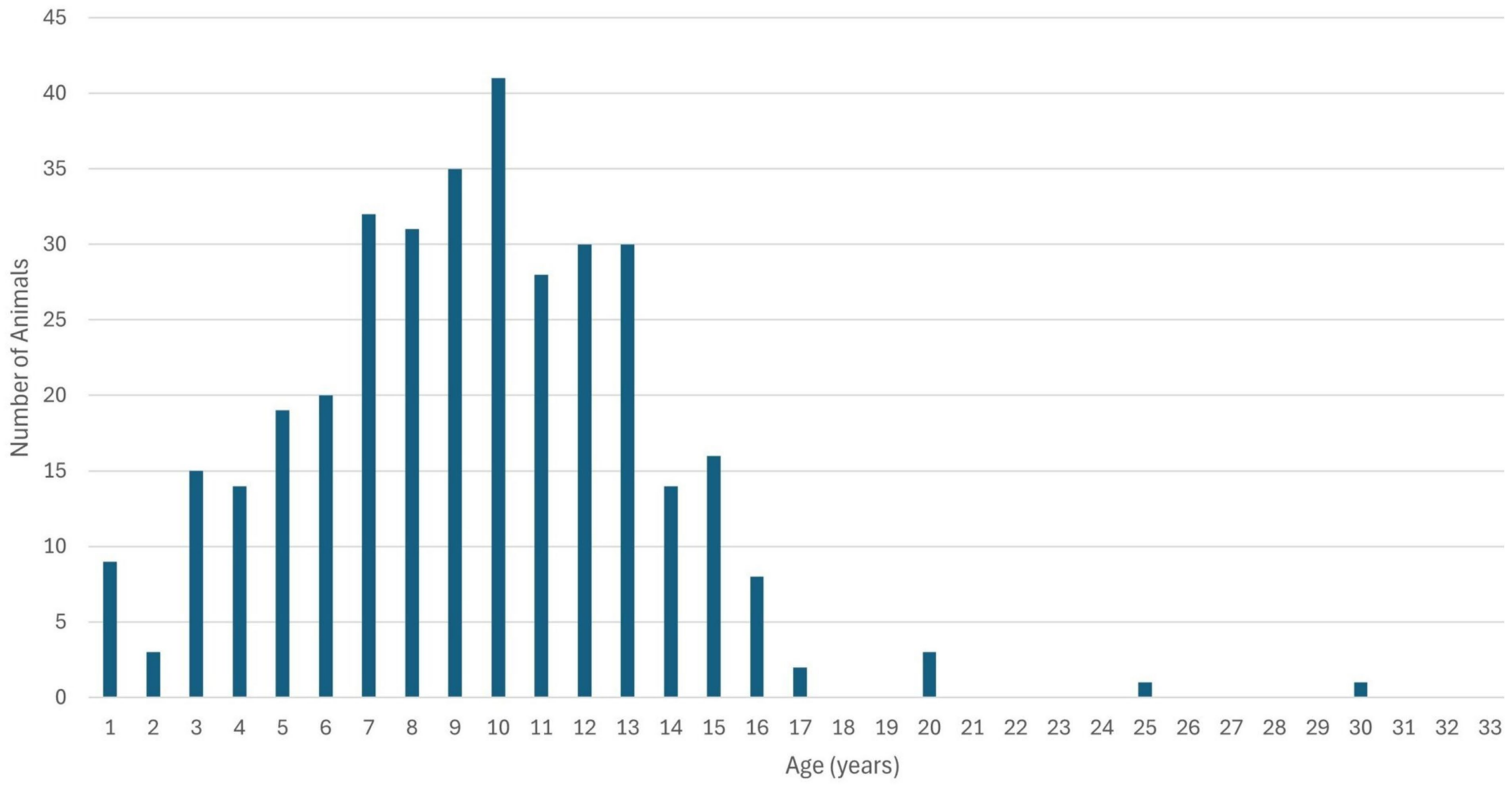
Boxplot of lesion evolution duration (days) by diagnostic groups. A significant difference was observed between the neoplasia and ‘other’ groups (*p* = 0.0068). Although the median lesion evolution time in the neoplasia group exceeded the third quartile of the other two groups, the upper quartile of the inflammation group extended long enough to prevent a statistically significant difference between the neoplasia and inflammation groups. This pattern was not observed in the ‘other’ group, which, due to the shorter lesion evolution time, showed a very evident difference.

In 195 cases, the duration of lesion evolution, as reported by the owners, ranged from 3 to 2,187 days. A statistically significant association was found between lesion duration and lesion type, specifically between the “neoplasia” group and the “other” group (*p* = 0.0068) (Figure 5). However, the sample sizes were unbalanced, with 164 cases in the “neoplasia” group and only 14 in the non-neoplastic group. The mean duration of lesion evolution in the neoplasia group was 266.2 days (SD = 384.2), with a median of 152 days. In the “inflammation” group, the mean duration was 134.2 days (SD = 177.5) and the median was 60 days. Among cases categorized as “other,” the mean duration was 83.14 days (SD = 71.27), with a median of 60 days.

### 3.7 Agreement between clinical and histopathologic diagnoses

Of the total cases, 239 included a clinical suspicion recorded on the examination request form, most of which (202/239) suspected of neoplastic disease. The overall agreement between clinical suspicion and histopathological diagnosis was 84.1%, compared with an expected agreement by 68.68%. The resulting Cohen’s Kappa coefficient was 0.492 (standard error = 0.062), indicating moderate agreement between clinical and histopathological assessments. The 95% confidence interval for the Kappa value ranged from 0.372 to 0.613, supporting its classification as moderate agreement according to the criteria proposed by Landis and Koch (5).

### 3.8 Species-specific lesion profiles

**Figure 6:**
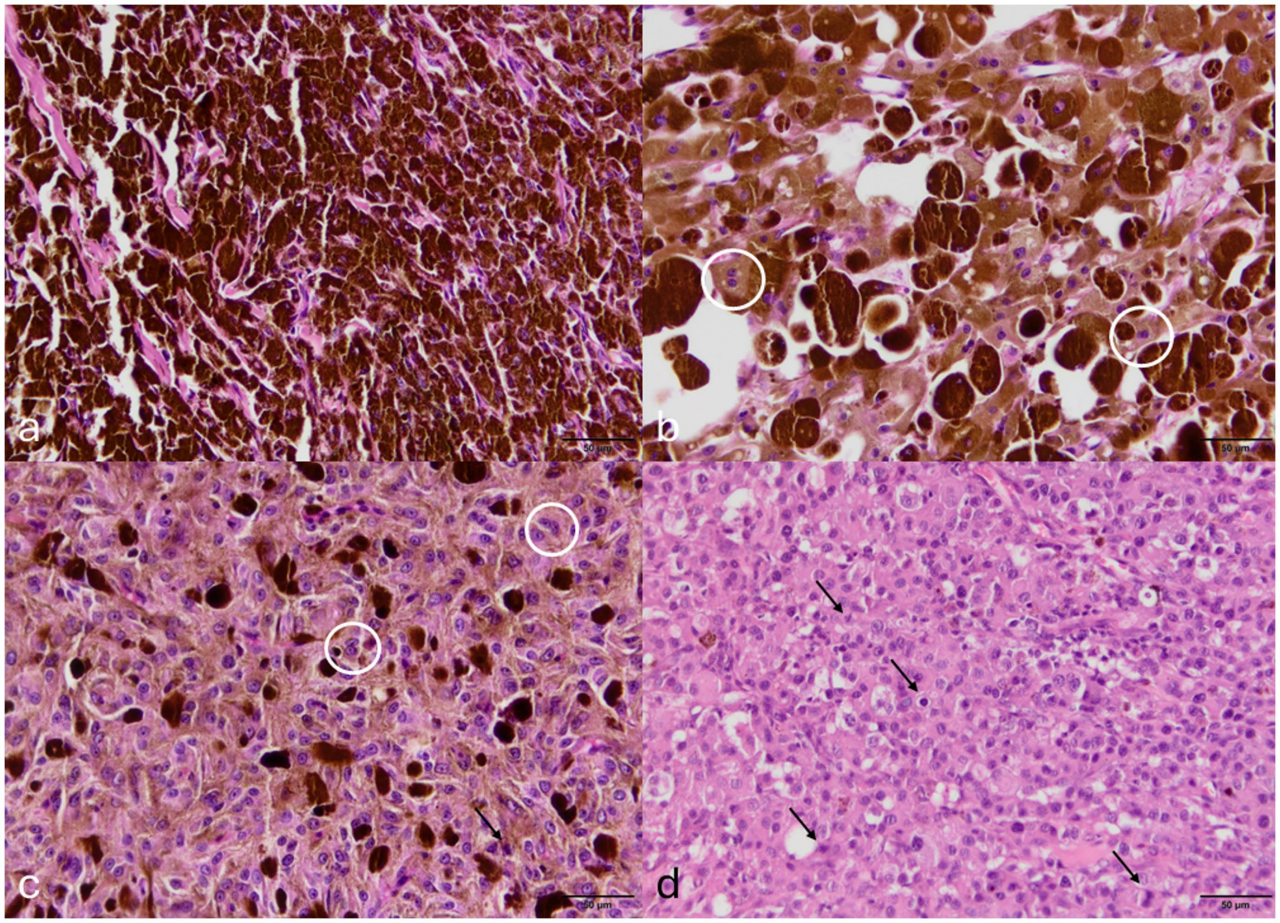
Canine uveal melanocytic neoplasms. **(A)** Melanocytoma: Highly pigmented neoplasm with low mitotic count and minimal malignancy criteria. Haematoxylin and eosin (HE). Bar, 50 μm. **(B)** Melanocytoma: Area showing a mild degree of cellular atypia, such as binucleation (circled), a feature that may mislead pathologists and result in melanoma overdiagnosis. HE. Bar, 50 μm. **(C)** Melanoma: Case with more evident cellular atypia, such as binucleation (circled) and variation in nuclear size, and reduced melanin content, but still with a low to moderate mitotic count (arrow). HE. Bar, 50 μm. **(D)** Melanoma: Marked reduction of melanin and high mitotic count (arrow) (diagnosis confirmed by immunohistochemistry). HE. Bar, 50 μm.

In dogs, the most frequently observed lesions were ocular melanoma (23 cases) (Figure 6) and neoplasms of the meibomian glands, which included 25 adenomas, 33 epitheliomas, and 15 adenocarcinomas. Twenty-six cases of meibomian gland neoplasia were associated with chalazion.

In cats, neoplastic lesions predominated, accounting for 71.92% of cases (41/57), with most classified as malignant (37/41). The most frequent lesions were SCC of the eyelid and conjunctiva, with 12 and four cases, respectively. Among the 28 conjunctival samples submitted, eight were from cats, four of which were diagnosed with SCC. One cat had a prior diagnosis of microphthalmia and underwent tarsorrhaphy approximately three years before tumor detection. After several drainages of serosanguineous fluid from the orbital cavity, the eyelids were surgically reopened, revealing a verrucous mass. This animal also had previous diagnoses of SCC affecting the ear pinna and nostril. The second animal underwent excision of a conjunctival growth for the third time after two recurrences at the same site. The third animal exhibited conjunctival edema and hyperemia approximately two months before diagnosis. No clinical history was available for the fourth cat.

A high frequency of SCC was observed among large animals, with 30/46 cases in horses, 13/15 in cattle, 3/4 in sheep, 2/2 in goats, and 1/2 in mules. Among the 16 equine globes submitted for analysis, 14 were diagnosed with SCC, primarily affecting the ocular surface (cornea and sclera). In cattle, the predominant site of SCC was the third eyelid. Of the 13 samples obtained from this site, 12 were diagnosed with SCC. The remaining bovine SCC case was located on the left upper eyelid.

## 4 Discussion

Of the 9,382 cases submitted for histopathological analysis during the study period, 418 (4.5%) were related to ocular pathology. This prevalence was significantly higher than previously reported by Hesse et al. (6) and Martins and Barros (2), who observed rates of 1.21 and 1.6%, respectively. Neoplastic lesions predominated, representing 80.53% of ocular cases. Other authors have also reported a higher number of submissions involving periocular or intraocular masses (1, 7). This trend likely reflects the clinical need for histopathological confirmation of proliferative lesions, which is critical for guiding therapeutic decisions (8). The prevalence observed in this study is similar to that reported by Martins and Barros (2), though both are substantially higher than rates reported elsewhere, which approximate 40% for neoplastic lesions (1, 7). Such variation may result from differences in sample submission protocols among institutions. In the present setting, most samples were referred by the Ophthalmology Sector, where clinicians with specialized ocular training are better able to identify and select cases requiring surgical intervention and histopathological examination. This targeted referral process likely contributed to the high proportion of neoplastic diagnoses observed.

The predominance of neoplastic submissions aligns with a general trend observed when comparing retrospective studies from veterinary ophthalmology clinics and pathology laboratories. Retrospective studies conducted in clinics often report a distribution of ocular lesions dominated by traumatic injuries and corneal alterations, with a lower incidence of neoplasms (9, 10). In contrast, studies based on pathology laboratory submissions consistently exhibit a marked predominance of neoplastic lesions (1, 2). This difference supports the explanation that specialized referrals to a pathology service prioritize cases requiring confirmation of proliferative disease, rather than representing the broader spectrum of cases typically seen in a general ophthalmology clinic.

Almost all cases exhibited some degree of inflammation. However, only lesions without a clearly identifiable etiology were classified as inflammatory, totaling 39 cases. Consistent with the neoplastic findings, the incidence of inflammatory lesions observed in this study aligns with that reported by Martins and Barros (2).

More than half of the samples analyzed originated from dogs, followed by cats. With minor regional variations, these species are the most commonly encountered in veterinary practice due to their close relationship with humans (1, 2, 11). This trend reflects the routine at the Veterinary Hospital in addition pathological diagnostic services rendered. Located in Brazil’s largest metropolis, this hospital primarily serves companion animals from the surrounding urban area, although referrals are also received from neighboring cities.

Regarding dogs, the predominance of samples from this species is consistent with findings from other studies (1, 2, 7, 11). However, in the study conducted by Martins and Barros (2), cattle represented the second most frequently sampled species. This difference likely reflects regional agricultural practices, as their study was conducted in southern Brazil, an area with a strong tradition of meat and wool production. Consequently, the higher presence of farm animals in that region leads to a greater representation of cattle in local veterinary hospitals caseloads.

A higher relative frequency of neoplastic lesions was observed in non-brachycephalic dogs. Although brachycephalic breeds are predisposed to ocular alterations due to their anatomical conformation, evidence linking this predisposition specifically to ocular neoplasms is limited (12, 13). The similarity between mean and median ages within the groups suggests a uniform distribution, indicating that age was not a confounding factor in the observed association. These findings suggest that brachycephalic dogs had a lower proportion of neoplastic lesions compared with non-brachycephalic dogs, independent of age. However, the limited sample size in the subgroup may have influenced the statistical results. Future studies with more balanced group sizes and consideration of additional variables, such as comorbidities, are recommended to validate and expand upon these findings.

The eyelids were the most frequently affected anatomical location, consistent with previous reports (1, 2). This region is highly visible and, in addition to being prone to proliferative lesions, is easily noticed by animal owners, increasing the likelihood of referred for veterinary evaluation.

The high frequency of SCC in the third eyelid of cattle observed in this study aligns with the findings of Martins and Barros (2). SCC is the most common tumor in cattle (14), and its development is strongly associated with ultraviolet (UV) radiation, a well-established predisposing factor (7, 15). Breeds such as Holstein cattle also exhibit unpigmented skin in mucocutaneous junctions, further increasing susceptibility to SCC (7, 14, 15). SCC was also the most frequently diagnosed tumor in both cats and horses, consistent with previous studies (2, 6, 16).

Although tumors predominated in the retrobulbar region (12/12) and eyelids (149/205), the statistical analysis revealed only a weak association between anatomical location and lesion type, despite statistical significance. This weak association suggests that, while certain sites may be more frequently affected and submitted for histopathology, other factors beyond location also play an important role in determining lesion type, such as environmental influences. These findings align with previous reports indicating that specific anatomical regions, particularly the eyelids, are more prone to SCC, largely due to increased exposure to UV radiation (7, 15).

Sex distribution varied among species, with a predominance of females, particularly in feline and bovine cases. All bovine samples were from female animals. However, no statistically significant association was found between sex and lesion type, suggesting that the observed distribution likely reflects a sampling bias common in retrospective studies. In cattle, this pattern is explained by dairy management practices, which primarily involve female animals. This aligns with the breed profile observed in this study (Holstein), as males are typically raised for meat production and slaughtered at an early age (17). Hesse et al. (6), despite focusing solely on ocular neoplasms, also reported a predominance of female cats, although no definitive explanation was provided.

The asymmetric age distribution aligns with the expected epidemiological profile of neoplastic lesions, which predominated in this study and generally occur more frequently in older animals (6). This pattern accounts for the significant age difference observed between the neoplastic and non-neoplastic groups. The latter includes lesions not typically associated with age, such as degenerative, infectious, and traumatic conditions, which tend to exhibit a more random age distribution (18, 19).

Although the data indicate a high level of agreement between clinical suspicion and the final histopathological diagnosis, interpretation using the Kappa coefficient and based on the widely adopted Landis and Koch (19) scale, reveals a moderate level of concordance. The Kappa value of 0.492, with a 95% confidence interval of 0.372–0.613, suggests that, in the worst-case scenario, agreement is weak to moderate, while in the best-case scenario it reaches moderate to substantial levels. Even though 84% of clinical suspicions matched histopathological findings, the adjusted Kappa indicates that a significant portion of this agreement could be attributed to chance. The results underscore a reasonable, though not definitive, alignment between clinical judgment and histopathological confirmation. This reinforces the necessity for diagnostic complementation, particularly in the evaluation of proliferative lesions, where histopathology remains essential for definitive characterization (7).

In this study, a high number of ocular melanomas (*n* = 23) and only one case of uveal melanocytoma were identified, contrasting with previous reports (7). Similar findings were described by Moreira et al. (1), suggesting that samples are predominantly submitted when lesions exhibit clinically aggressive behavior. It is important to note, however, that even benign intraocular tumors can produce malignant clinical signs, primarily due to disruption of aqueous humor outflow, which may lead to secondary glaucoma (2). Another possible explanation for the observed discrepancy is overdiagnosis of uveal melanomas. This is supported by the increase in diagnosed malignant neoplasms without a corresponding rise in clinical outcomes, such as mortality (20). The implementation of standardized diagnostic criteria through established guidelines has proven effective for other tumor types, including cutaneous melanomas, and may help reduce diagnostic variability and improve consistency across pathologists (20, 21).

Among the eyelid neoplasms identified in this study, 73.4% (69/94) originated from the meibomian gland. These lesions represent 28.75% (69/240) of all canine samples, a frequency similar to that reported by Martins and Barros (2). Meibomian adenocarcinomas are generally considered rare, and the incidence observed here exceeds expectations based on the literature (7). This discrepancy raises the possibility of overdiagnosis, emphasizing the need for re-evaluation using well-established diagnostic criteria (20).

Chalazion is frequently associated with meibomian neoplasms and is histologically characterized by epithelioid macrophages, multinucleated giant cells, and lipid-laden debris (7). It may also occur as a primary lesion without an associated tumor (7), as observed in a single case in this study. Among the 15 meibomian adenocarcinomas identified, nine were associated with chalazion, a finding more commonly reported in benign lesions (7).

Despite the predominance of malignant tumors in cats, four benign lesions were identified. These included an apocrine cystadenoma, an apocrine ductular adenoma, and a fibroma in the eyelid, as well as an apocrine ductular adenoma in the periocular region. These findings contrast with those of Martins and Barros (2), who did not report any benign tumors in this anatomical region in cats.

Conjunctival alterations in cats are relatively common, primarily due to infectious agents in the region, which often result in conjunctivitis (22). However, conjunctival SCC in cats is considered extremely rare (7), a finding not supported by the present study. In this survey, half of the feline conjunctival samples were diagnosed as SCC. One might hypothesize that these neoplasms represent extensions of tumors originating from adjacent anatomical structures, where SCC is more prevalent. However, among the three cats with available clinical histories, all cases were clearly described as originating in the conjunctiva itself. One of these animals had a documented history of SCC in other anatomical locations, suggesting the possibility of multicentric SCC, which may increase the likelihood of conjunctival involvement (7).

This retrospective study, spanning two decades, revealed a wide spectrum of morphological alterations affecting the ocular and periocular regions, with a clear predominance of neoplastic lesions. This pattern underscores the critical role of histopathological evaluation as a definitive diagnostic method. Increased submission of ocular and periocular samples for histopathology is essential, not only to enhance understanding of the epidemiological patterns of these lesions but also to provide veterinary pathologists with diagnostically challenging cases, thereby contributing to diagnostic refinement and professional development.

## Data Availability

The original contributions presented in the study are included in the article, further inquiries can be directed to the corresponding author/s.
